# Potential risk for dengue hemorrhagic fever: the isolation of serotype dengue-3 in Mexico.

**DOI:** 10.3201/eid0202.960210

**Published:** 1996

**Authors:** B Briseño-García, H Gómez-Dantés, E Argott-Ramírez, R Montesano, A L Vázquez-Martínez, S Ibáñez-Bernal, G Madrigal-Ayala, C Ruíz-Matus, A Flisser, R Tapia-Conyer

**Affiliations:** Instituto Nacional de Diagnóstico y Referencia Epidemiológicos, Dirección General de Epidemiología, Secretaría de Salud, Mexico D.F., Mexico.


### Potential Risk for Dengue Hemorrhagic Fever: The Isolation of Serotype Dengue-3 in Mexico

The Americas have a long history of dengue epidemics, which present public health problems because of the potential emergence of dengue hemorrhagic fever (DHF) (1). Efforts to control *Aedes aegypti*—the only demonstrated vector of dengue virus in the Americas—were effectively deployed in the 1950s and 1960s when the Pan American Health Organization launched a continental eradication campaign against yellow fever (2). *Aedes aegypti* was eliminated in Mexico in 1963 (3). However, subsequent social and economic changes in the Americas have permitted the rapid reinfestation of the vector throughout the region. In Mexico, population movement from rural areas to urban centers—brought about by intensive industrialization—were not matched with adequate housing and sufficient water, sewage, and waste management systems. The introduction and proliferation of nonrecyclable products provided numerous and effective breeding sites for urban mosquitoes. For example, from 1960 to 1990, the annual production of bottles in Mexico increased from 500,000 to 3.5 million, and the annual production of tires increased from 2 to 17 million (4). Tourism and travel, promoted as essential to the national economy, have also become important mechanisms for transporting dengue viruses. Additionally, surveillance, prevention, and control programs lack the infrastructure and human resources needed to tackle this neglected health problem (1,4). Millions of people living in the tropical and subtropical areas of the region face the reemergence of dengue and DHF (2).

In Mexico from 1984 to 1993, DHF cases were sporadically reported; only 26 cases were identified, followed by 30 cases in 1994 (4). During 1995, however, the General Directorate of Epidemiology of the Ministry of Health in Mexico confirmed 358 DHF cases in 18 states with a case-fatality rate of 7.8% (unpublished data). The widespread distribution of DHF cases and of the vector and the circulation of different serotypes demonstrate the risk of serious illness throughout the country.

Dengue fever in endemic-disease areas is often not diagnosed properly because of its nonspecific clinical manifestations. Furthermore, only patients with symptoms are treated, and patients rarely demand medical care; thus, the proportion of infected persons in the population is usually underestimated (5). On the other hand, DHF is an acute, life-threatening disease that requires specialized treatment in a medical setting. Identifying dengue serotypes in the continent is one of the most serious problems faced by every surveillance system in the region. The serotype, strain, and sequence of infections by different serotypes are among the most meaningful risk factors for DHF; thus, creating a strong dengue virus surveillance system in every country in the Americas should be a high priority (6, 7).

Serologic evidence of dengue in the Americas can be traced back to 1941 in Panama (8). DEN-2 was isolated in Trinidad in 1953 (9). DEN-3 was isolated in the Caribbean and Venezuela in 1963 (2,10), DEN-1 was introduced to the Americas in 1977, and DEN-4 affected the region 4 years later. In 1981, Cuba had a major DHF epidemic caused by a new strain of DEN-2 (11). DEN-3 was detected in Nicaragua and Panama in 1994 and in Costa Rica in 1995 (12), after a long absence from the region; a strain similar to one in Sri Lanka and India in the 1980s caused the DHF epidemics in those countries (12). The identification of DEN-3 in the region increases the probability of DHF cases associated with a newly circulating serotype. In Mexico, this particular situation may have important epidemiologic consequences for several reasons: 1) DEN-3 has not been identified in the country, and the population is totally susceptible to infection by this serotype; 2) infection by DEN-3 would most likely be of the secondary type; 3) population movements through Mexico and towards other countries, might disseminate this new serotype to areas where susceptible persons will be exposed to a new serotype; and 4) intensive transmission of dengue would naturally increase the risk for DHF epidemics.

Surveillance of dengue virus in Mexico began in 1982 when seven isolates of DEN-1 and DEN-2 were identified from outbreaks reported in the south and southeastern regions of the country. From 1982 to 1995, the National Institute of Epidemiological Diagnosis and Reference (INDRE) identified 681 dengue virus isolates. Serotypes were identified by indirect immunofluorescence with specific monoclonal antibodies donated by the Division of Vector-Borne Infectious Diseases, Centers for Disease Control and Prevention, Fort Collins, Colorado.

DEN-1 was the serotype most frequently isolated from 1982 to 1995 (47% of all isolates), followed by DEN-4 (30%) and DEN-2 (21%) (Table). In 1995, DEN-3 was identified in 19 patients with classic dengue fever with no hemorrhagic manifestations (Table). Beginning in 1995, active surveillance for dengue cases was begun in areas where transmission had been documented. Sentinel surveillance centers were implemented to obtain serum samples from febrile patients with a clinical picture suggestive of dengue and to isolate and identify the serotype involved. From August to December 1995, 245 isolates of dengue virus were obtained, which represented 36% of isolates obtained during the 14-year period. The prevalence of serotypes isolated in 1995 differed from those isolated from 1982 to 1994; DEN-1 was isolated in only 16% of the samples processed, whereas 40% were DEN-2, 8% were DEN-3, and 36% were DEN-4 (Figure 1). It is unclear whether the change in distribution of serotypes is due to more intensive surveillance in certain areas or in a manifestation of herd immunity to serotype 1. This is the first report of DEN-3 in Mexico and reflects the strengthening of the surveillance at INDRE for dengue viruses in areas at risk.

The geographic and temporal distribution of DEN-3 isolated in 1995 in Mexico (Figure 2) shows a pattern similar to the one followed by the first dengue epidemics in the early 1980s (2) and may be related to population movements towards the northern border. The role of DEN-3 in increasing DHF cases is still to be determined; to date none of the DHF cases in which dengue virus was isolated have been associated with this serotype. Five cases were associated with DEN-1 and 20 cases with DEN-2. Nevertheless, the infection of DEN-3 in persons sensitized by previous infections with other serotypes and the widespread susceptibility of the Mexican population to this serotype must be considered a potential risk factor for future outbreaks.

The cost of each DHF case has not been fully determined, but the resources needed to face a DHF epidemic are certainly not available in countries where the health sector has financial constraints due to unstable economic conditions. The development of dengue vaccines is encouraging, but the widespread dispersion of mosquito breeding sites exceeds the capabilities of vector control programs. Moreover, the potential role of

Table. Number of isolates of dengue virus serotypes in Mexico*

| Year | DEN-1 | DEN-2 | DEN-3 | DEN-4 | Total |
|---|---|---|---|---|---|
| 1982 | 2 | 5 | 0 | 0 | 7 |
| 1983 | 5 | 6 | 0 | 2 | 13 |
| 1984 | 89 | 2 | 0 | 38 | 129 |
| 1985 | 30 | 8 | 0 | 9 | 47 |
| 1986 | 65 | 0 | 0 | 24 | 89 |
| 1987 | 13 | 0 | 0 | 0 | 13 |
| 1988 | 28 | 0 | 0 | 0 | 28 |
| 1989 | 21 | 0 | 0 | 0 | 21 |
| 1990 | 6 | 0 | 0 | 0 | 6 |
| 1991 | 4 | 0 | 0 | 20 | 24 |
| 1992 | 1 | 5 | 0 | 19 | 25 |
| 1993 | 0 | 10 | 0 | 0 | 10 |
| 1994 | 15 | 9 | 0 | 0 | 24 |
| 1995 | 40 | 98 | 19 | 88 | 245 |
| Total | 319 | 143 | 19 | 200 | 681 |

*Serum samples from suspect cases were added to C6-36 cells grown in D-MEM with 5% fetal calf serum for 7 days at 28° C and incubated for 1 hour. Cells were washed and further incubated in D-MEM with 0.4% bovine albumin for identification of cytopathic effect.

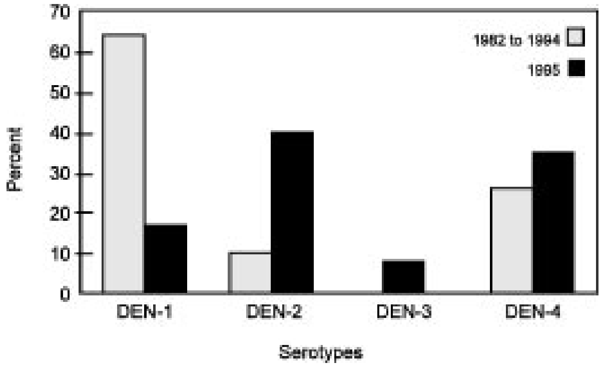

Figure 1. Frequency of dengue serotypes isolated in 1982 to 1994 and in 1995.

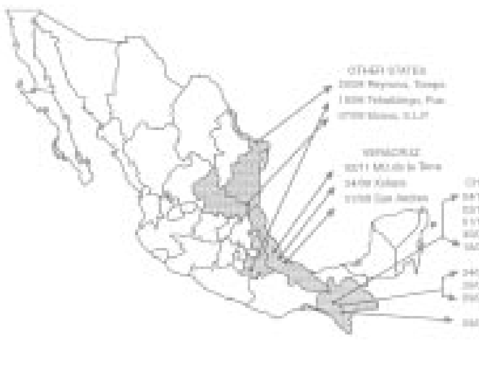

Figure 2. Geographic and temporal distribution of DEN-3 serotype in Mexico.

*Aedes albopictus* in the transmission of dengue virus in Mexico must be evaluated because DHF cases have appeared in areas where *A. albopictus* has been identified (14). The role of this vector in dengue transmission could increase should its geographic distribution expand and its susceptibility to infection increase (15).

The challenge faced by national health services is to improve the early detection of dengue transmission, prevent DHF, and decrease the case-fatality rate in DHF patients. This strategy must be supported by a strong surveillance network for viral diseases, which is now being implemented on a regional basis according to the risk of dengue transmission in the country. The detailed knowledge of the serotypes involved in future epidemics will provide useful information that will define the role of each serotype in the genesis of DHF cases and target control measures. The threat of a major epidemic requires a control strategy that will prevent the emergence of this public health problem.

**Baltasar Briseño-García,* Héctor Gómez-Dantés,†‡ Enid Argott-Ramírez,* Raul Montesano, Ana-Laura Vázquez-Martínez,* Sergio Ibáñez-Bernal,* Guillermina Madrigal-Ayala,* Cuauhtémoc Ruíz-Matus,† Ana Flisser,* and Roberto Tapia-Conyer†**

*Instituto Nacional de Diagnóstico y Referencia Epidemiológicos, Dirección General de Epidemiología, Secretaría de Salud, México D.F., México; †Dirección General de Epidemiología, Secretaría de Salud, México D.F., México; ‡Present address: Centro de Investigación de Enfermedades Infecciosas, Instituto Nacional de Salud Pública, Secretaría de Salud, Cuernavaca, México

### References

1. Gubler DJ, Trent DW. Emergence of epidemic dengue/dengue hemorrhagic fever as a public health problem in the Americas. Infect Agents Dis 1993; 2: 383-393.
2. Pan American Health Organization. Dengue and dengue hemorrhagic fever in the Americas: Guidelines for prevention and control. Scient Publ 1994;548:3-22.
3. Torres-Muñoz A. La fiebre amarilla en Mexico: erradicación de *Aedes aegypti*. Salud Publica Mex 1995;37:S103-S110.
4. Narro RJ, Gómez-Dantés H. El dengue en Mexico: un problema prioritario de salud pública. Salud Publica Mex 1995; 37:S12-S20.
5. Dietz VJ, Gubler DJ, Rigau-Pérez J, Pinheiro F. Epidemic of dengue 1 in Brazil, 1986: evaluation of a clinically based dengue surveillance system. Am J Epidemiol 1990;131:693-701.
6. Gubler DJ. Vigilancia activa del dengue y de la fiebre hemorrágica del dengue. Bol Of Sanit Panam 1989;107:22-30.
7. Rico-Hesse R. Molecular evolution and distribution of dengue virus type 1 and 2 in nature. Virology 1990;174:479-93.
8. Rosen L. Observations on the epidemiology of dengue in Panama. Am J Trop Med Hyg 1958;68: 45-58.
9. Anderson CR, Downs WG, Hill AE. Isolation of dengue virus from a human being in Trinidad. Science 1956;124:224-5.
10. Pinheiro F. Dengue in the Americas 1980-1987. PAHO Epidemiol Bull 1989;10:1-7.
11. Gubler D. Dengue/dengue hemorrhagic fever in the Americas: prospects for the year 2000. In: Halstead SB, Gomez-Dantes H, editors. Dengue: a worldwide problem, a common strategy. Proceedings of the International Conference on Dengue and *Aedes aegypti* community-based control, Merida, Mexico, July 11-16, 1992; Ministry of Health, Mexico,1992:19-27.
12. Gubler JD, Clark GG. Dengue/dengue hemorrhagic fever: the emergence of a global health problem. Emerging Infectious Diseases 1995;1:55-7.
13. Gubler DJ. *Aedes aegypti* and *Aedes aegypti*-borne disease control in the 1990's: top down or bottom up. Am J Trop Med Hyg 1989;40: 571-8.
14. Ibañez-Bernal S, Martínez-Campos C. *Aedes albopictus* in Mexico. J Am Mosq Control Assoc 1994; 10: 231-2.
15. Shope R. Global climate change and infectious diseases, Environ Health Perspect 1991;96:171-4.
